# Supplementary material for: Whole-Body MRI Surveillance—Baseline Findings in the Swedish Multicentre Hereditary TP53-Related Cancer Syndrome Study (SWEP53)
Source: Cancers (Basel). 2022 Jan 13;14(2):380. doi: 10.3390/cancers14020380 (PMC8773910; doi:10.3390/cancers14020380)
Supplement: Supplementary file 1 [file cancers-14-00380-s001.zip › cancers-1523821-supplementary.pdf]

# Supplementary material: Whole-body MRI Surveillance – Baseline findings in a Swedish multicentre Hereditary *TP53*-related cancer syndrome study (SWEP53)

**Meis Omran, Emma Tham, Yvonne Brandberg, Håkan Ahlström, Claudia Lundgren, Ylva Paulsson-Karlsson, Ekaterina Kuchinskaya, Gustav Silander, Anna Rosén, Fredrik Persson, Henrik Leonhardt, Marie Stenmark-Askmalm, Johanna Berg, Danielle van Westen, Svetlana Bajalica-Lagercrantz and Lennart Blomqvist.**

**Supplement S1**

**Table S1. Imaging protocol, WB-MRI.**

| Coverage | Pulse sequence | Imaging plane | Comment |
|---|---|---|---|
| **Whole-body from skull base** | | | |
| | T2-weighted turbo spin-echo | Transaxial | Half fourier single shot turbo spin echo |
| | T1-weighted gradient echo | Transaxial | Dixon based |
| | Diffusion-weighted | Transaxial | Echo-planar based |
| **Brain** | | | |
| | T1-weighted gradient echo | Transaxial | Three-dimensional acquisition (3D) |
| | Fluid attenuated inversion recovery | Sagittal | 3D FLAIR |
| **Breast** | | | |
| | Inversion recovery | Sagittal | Turbo spin echo based |
| | T1-weighted gradient echo | Transaxial | 3D Refocused gradient echo |
| | T2-weighted | Transaxial | Dixon based |
| | Diffusion-weighted | DWI | Echo-planar based |
| | T1-weighted gradient echo | Transaxial | 3D Refocused gradient echo with intravenous Gadolinium chelate |
